# Postglacial Colonisation Patterns and the Role of Isolation and Expansion in Driving Diversification in a Passerine Bird

**DOI:** 10.1371/journal.pone.0002794

**Published:** 2008-07-30

**Authors:** Bengt Hansson, Dennis Hasselquist, Maja Tarka, Pavel Zehtindjiev, Staffan Bensch

**Affiliations:** 1 Department of Animal Ecology, Lund University, Ecology Building, Lund, Sweden; 2 Institute of Zoology, Bulgarian Academy of Sciences, Sofia, Bulgaria; University of Kent, United Kingdom

## Abstract

Pleistocene glacial cycles play a major role in diversification and speciation, although the relative importance of isolation and expansion in driving diversification remains debated. We analysed mitochondrial DNA sequence data from 15 great reed warbler (*Acrocephalus arundinaceus*) populations distributed over the vast Eurasian breeding range of the species, and revealed unexpected postglacial expansion patterns from two glacial refugia. There were 58 different haplotypes forming two major clades, A and B. Clade A dominated in Western Europe with declining frequencies towards Eastern Europe and the Middle East, but showed a surprising increase in frequency in Western and Central Asia. Clade B dominated in the Middle East, with declining frequencies towards north in Central and Eastern Europe and was absent from Western Europe and Central Asia. A parsimonious explanation for these patterns is independent postglacial expansions from two isolated refugia, and mismatch distribution analyses confirmed this suggestion. Gene flow analyses showed that clade A colonised both Europe and Asia from a refugium in Europe, and that clade B expanded much later and colonised parts of Europe from a refugium in the Middle East. Great reed warblers in the eastern parts of the range have slightly paler plumage than western birds (sometimes treated as separate subspecies; *A. a. zarudnyi* and *A. a. arundinaceus*, respectively) and our results suggest that the plumage diversification took place during the easterly expansion of clade A. This supports the postglacial expansion hypothesis proposing that postglacial expansions drive diversification in comparatively short time periods. However, there is no indication of any (strong) reproductive isolation between clades and our data show that the refugia populations became separated during the last glaciation. This is in line with the Pleistocene speciation hypothesis invoking that much longer periods of time in isolation are needed for speciation to occur.

## Introduction

Pleistocene glacial cycles cause repeated range expansions and contractions with important present-day demographic consequences for temporal and boreal populations and species in the Northern Hemisphere [1]–[3]. When populations are separated and isolated in glacial refugia for a sufficient period of time they may evolve pre- or post-zygotic reproductive barriers and become reproductively isolated sister taxa [4]–[6]. Following postglacial range expansions such taxa may, depending on the degree of diversification, form hybrid zones with at least some interbreeding and introgression, and reinforcement processes may complete the speciation event [7]–[9]. Assuming a constant mitochondrial molecular clock whereby substitutions take place at a rate of 1% per Myr, genetic distances between sister taxa in birds at higher latitudes translate to divergence times of approximately 0.1–3.0 Myr. As formulated in the “Pleistocene speciation hypothesis”, a common view is that these speciation events took place in isolated refugia over one to several full glacial cycles [4]–[6].

An alternative hypothesis, referred to as the “postglacial expansion hypothesis”, is that speciation takes place during expansion rather than during isolation [10]–[13]. During rapid postglacial range expansions advancing populations encounter a wide variety of unoccupied habitats with varying selection regimes, which could drive diversification in comparatively short periods of time. Since this scenario does not invoke long-term isolation in refugia, the postglacial expansion hypothesis poses an alternative to traditional models of Pleistocene speciation. In support of the postglacial expansion hypothesis, recent divergence with extensive morphological variation has, for example, been documented in several bird species [10]–[13].

Here we use molecular genetic data to evaluate postglacial colonization patterns in a bird with only slight phenotypic variation, the great reed warbler (*Acrocephalus arundinaceus*). This is a socially polygynous long-distance migratory passerine bird that breeds in reed marsh habitats in most of the Palaearctic between latitudes 35° and 60° N, east to North-Western Mongolia [14]–[16]. Great reed warblers in the eastern parts of the range have slightly paler plumage than western birds and sometimes two subspecies are recognised, *A. a. arundinaceus* in the western parts, and *A. a. zarudnyi* in the eastern parts of the range [14], [17]. In a previous study, Bensch and Hasselquist [18] looked into the phylogeography of the species in the western part (Western and Central Europe) of the breeding range. Interestingly, they found that great reed warblers had two main mitochodria DNA (mtDNA) haplotype clades (called A and B), where clade A occurred in all populations with declining frequencies towards east, whereas clade B was absent from Western Europe [18]. Based on these patterns it was hypothesised that birds with clade A haplotypes expanded from a glacial refugium in western parts and clade B from a refugium in the eastern parts of the species' breeding range [18].

In the present study we reveal a more complex colonization history of the great reed warbler (i) by using mtDNA sequence data from a total of 15 populations distributed over most of the species' Eurasian breeding range now including also Eastern Europe, the Middle East and Western and Central Asia, and (ii) by using recently developed statistical tools that make it possible to test more sophisticated hypotheses regarding the divergence and expansion, e.g. inferring asymmetric gene flow between populations and time since divergence [19], [20]. We document independent postglacial expansion events from two glacial refugia, where birds from a refugium in Europe (carrying clade A haplotypes) colonised not only Europe, but also Asia, before birds from a refugium in the Middle East (carrying clade B haplotypes) expanded north in Central and Eastern Europe. The consequences of repeated range expansions and contractions in general, and their role in driving diversification and speciation, are discussed.

## Results

### Haplotype and nucleotide diversity

We sequenced 494 bps of the control region II in 281 great reed warblers from 15 Eurasian populations (Table 1). This region contained 39 variable sites which defined 58 different haplotypes separated by up to 2.0%. Haplotype and nucleotide diversity for all samples were 0.913 (ranged between 0.286 for Iran and 0.947 for Hungary) and 0.00735 (ranged between 0.00117 for Iran and 0.00912 for the Czech Republic), respectively (Table 1).

**Table 1:** Sampling localities, sampling period, number of sequences (*n*), number of haplotypes (*nH*), haplotype diversity (*H*) and nucleotide diversity (*π*).

Country	Site	Coordinates	Sampling year	*n*	*nH*	*H*	*π*
Spain	Hondo Natural Park, Alicante	38°12′N, 0°42′W	1996	11	5	0.709	0.00202
The Netherlands	Zwarte Meer,Weerribben	52°37′N, 5°55′E	1995	10	4	0.533	0.00205
Sweden	Kvismaren, Närke	59°10′N, 15°25′E	1987–1990	22	7	0.649	0.00480
Latvia	Engure/Kanieris, Tukums	57°07′N, 23°20′E	1992	20	11	0.916	0.00654
Germany	Müggelsee, Berlin	52°26′N, 13°39′E	1992,1993	19	12	0.918	0.00652
The Czech Republic	Hodonin fishponds, Moravia	48°54′N, 17°02′E	2006	17	11	0.934	0.00912
Hungary	Apaj Channels, Kiskunlachaza	47°07′N, 19°05′E	2000	20	14	0.947	0.00565
Belarus	Turov, Zhitkovichi	52°01′N, 27°49′E	2000	17	9	0.890	0.00737
Ukraine	Usovka, Poltava	50°19′N, 32°32′E	2000	18	11	0.941	0.00799
	Denisyvka, Poltava	49°53′N, 32°36′E					
	Vilkovo, Odessa	45°28′N, 29°35′E					
Russia	Steppe liman, Saratov	50°43′N, 46°27′E	2006	28	12	0.857	0.00524
	Solyanka, Saratov	50°49′N, 47°05′E					
	Furmanovo, Saratov	51°38′N, 49°07′E					
Kazakhstan	Stone Lake, Zhambyl	42°49′N, 70°56′E	2001	35	9	0.793	0.00275
	Lake Balkhash, Almaty	45°12′N, 73°59′E	2006				
Greece	Limni Mikri Prespa, Florina	40°50′N, 21°05′E	1990	20	13	0.932	0.00906
	Mitrikou Lake, Rodopi	40°58′N, 25°17′E	2005				
Bulgaria	Kalimok, Tutrakan	44°01′N, 26°26′E	2005–2006	20	12	0.900	0.00836
Turkey	Mogan Lake, Ankara	39°46′N, 32°48′E	2005	17	7	0.824	0.00374
Iran	Zarin Kola, Mazandaran	36°44′N, 53°00′E	2004	7	2	0.286	0.00117
*Pooled*				*281*	*58*	*0.913*	*0.00735*

### Haplotype relationship and population divergence

**Figure 1:**
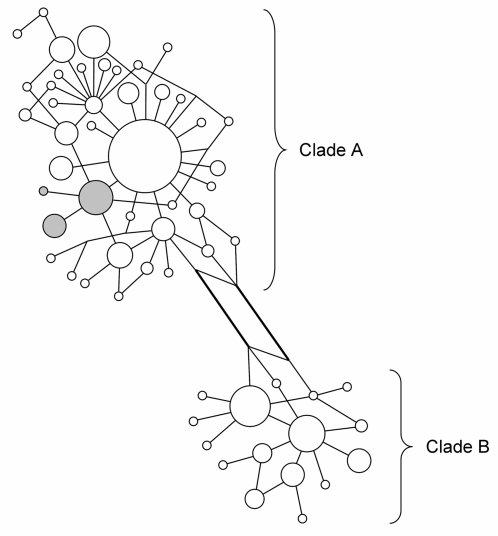
Minimum-spanning network (MSN) for 58 mtDNA control region II haplotypes in great reed warblers from 15 breeding populations. Circles are proportional to sample size (281 sequences in total) and each line corresponds to one substitution; with the exception of thicker lines, which correspond to two substitutions. The two major haplotype clades are shown, clade A and B. Grey shading indicates a relatively large uniform cluster of sequences within clade A only found in Russia and Kazakhstan (see text and Figure S1 for details).

A Neighbour-Joining analysis of the 281 sequences revealed two major clades (A and B) supported by a bootstrap value of 75% (Figure S1), and a minimum-spanning network (MSN) was drawn to show the relationship between haplotype within and between clades (Figure 1).

The average number of nucleotide substitutions per site between the clades, *D*_XY_, was 0.013 (±0.004 SE) and the number of net nucleotide substitutions per site between clades, *D*_A_, was 0.010 (±0.004 SE). Assuming a mutation rate of 15% (range 10–20%) per site Myr^−1^ for the control region [13], [21]–[24], a constant molecular clock and a generation time of 2 years [18], we obtain a divergence time for the two clades of 87 (65–141) and 65 (49–98) kyr BP for *D*_XY_ and *D*_A_, respectively.

**Figure 2:**
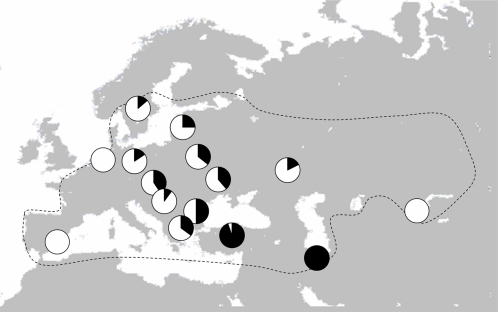
The frequencies of the two clades in each of the 15 great reed warbler breeding populations (clade A is in white and clade B in black). Breeding distribution is indicated (dotted line).

In Figure 2, we have plotted the frequencies of the two clades in each population. Clade A is more widely dispersed and dominates in most parts of the breeding range except in some populations in the Central Europe where it occurs in intermediate frequencies, and in the Middle East where it is almost absent (Figure 2). Clade B is more restricted geographically, and dominates in the Middle East with declining frequencies towards north in South-Eastern and Central Europe (Figure 2).

**Figure 3:**
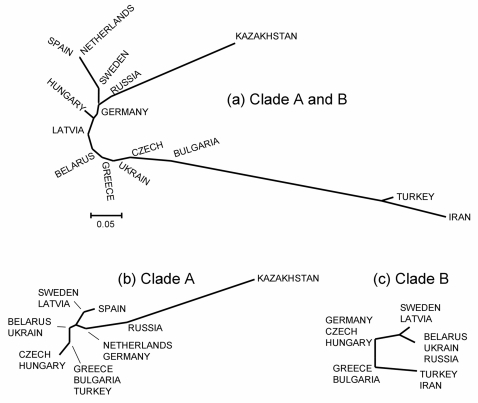
Neighbour-Joining (NJ) population trees generated from pair-wise population distances (*F*_ST_-values) for (a) all haplotypes, (b) clade A haplotypes, and (c) clade B haplotypes. Because of low samples sizes in some populations, we pooled closely located populations in the clade specific analyses (b and c).

The differentiation between pairs of populations measured as *F*_ST_ varied substantially and reached its highest value between the Netherlands and Iran (*F*_ST_ = 0.871; *F*_ST_-values are given in Table S1a). To illustrate the genetic distance between populations we generated a Neighbour-Joining (NJ) population tree from these pair-wise distances (Figure 3a). In this tree, three main branches could be seen: one leading to Spain and the Netherlands, another to Kazakhstan and the last one to Turkey and Iran. Thus, the main features of this tree correspond to the geographical distribution of clade A and B, although the two separate branches leading to Spain and the Netherlands, and Kazakhstan, respectively, suggest that there is also detectable population divergence within clade A.

To evaluate the latter, we performed separate analyses for sequences belonging to clade A and B. The sample size of each clade was low in some populations and therefore we pooled data from some closely located populations in these analyses. We used eight populations for clade A (Table S1b) and five populations for clade B (Table S1c). There were several high *F*_ST_-values for clade A, with the highest *F*_ST_ of 0.349 for Spain and Kazakhstan (Table S1b). The highest *F*_ST_-value for the clade B was 0.238 between Belarus/Ukraine/Russia and Turkey/Iran (Table S1c). In the corresponding NJ population tree for clade A, there was a long branch separating Kazakhstan from the other populations via Russia, and an additional shorter branch leading to Spain (Figure 3b). The NJ population tree for clade B showed overall shorter branches (Figure 3c).

A closer inspection of the NJ sequence tree reveals that there is a large relatively uniform cluster of 26 sequences from Kazakhstan and Russia in the central part of the tree (indicated with a dotted line in Figure S1; shown in grey in Figure 1), which explains the long branch leading to Kazakhstan in the NJ population trees (Figure 3a,b).

### Mismatch distributions and clade specific analyses of gene flow

**Figure 4:**
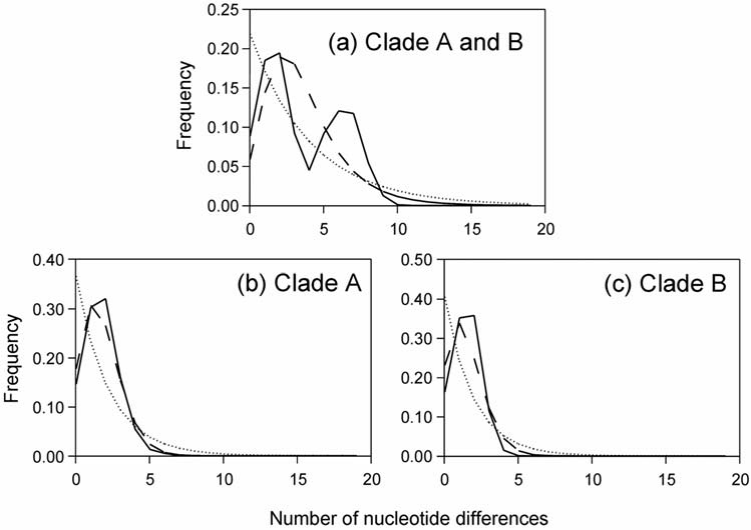
Mismatch distributions for (a) all haplotypes, (b) clade A haplotypes and (c) clade B haplotypes. Shown are the observed distribution (lines), the expected distribution of a population with constant size (dotted) and the expected distribution of an expanding population (dashed).

When all haplotypes were tested simultaneously there were two clear peaks in the mismatch distribution at 1–2 bp and 6–7 bp, respectively (Figure 4a). This distribution did not correspond to what would be expected in a stable population (χ^2^ = 67.0, df = 10, p<0.001) or an expanding population (χ^2^ = 48.5, df = 9, p<0.001). This supports the view that there have been expansions from two glacial refugia populations. When the two clades were tested separately, the mismatch distributions for both clades differed significantly from what would be expected in a stable population (clade A: χ^2^ = 49.2, df = 6, p<0.001; clade B: χ^2^ = 23.5, df = 4, p<0.001), whereas the distributions did not deviate from the expected in an expanding population (clade A: χ^2^ = 2.6, df = 5, p = 0.77; clade B: χ^2^ = 3.5, df = 3, p = 0.31) (Figure 4b,c). The value *τ* was 1.734 for clade A and 1.467 for clade B, which translate to dates of expansion of 23 (18–35) and 20 (15–30) kyr BP, respectively.

**Figure 5:**
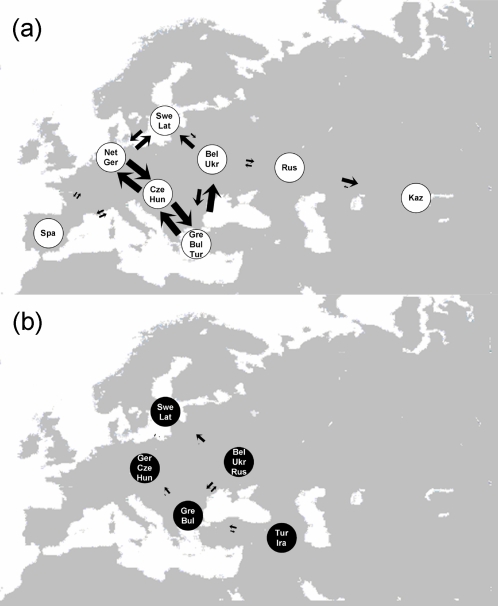
Schematic illustration of the gene flow between populations estimated with the IMa method for (a) clade A and (b) clade B. Size of arrows are proportional to the gene flow (the largest arrow gives M≥100 migrants per generation; see Table S2 for details). Labels refer to population (SPA–Spain; NET–the Netherlands; GER–Germany; SWE–Sweden; LAT–Latvia; BEL–Belarus; UKR–Ukraine; CZE–the Czech Republic; HUN–Hungary; BUL–Bulgaria; GRE–Greece; RUS–Russia; KAZ–Kazakhstan; TUR–Turkey; IRA–Iran).

We inferred the colonisation history of each clade separately by using isolation with migration models implemented in the program IMa [19], [20]. To increase sample size in each group, we pooled populations as in the previous analyses. For clade A, the present-day effective population sizes were approximately 50,000, the ancestral population size approximately 10,000 and the time since divergence between pairs of populations approximately 4 kyr (Table S2). The gene flow varied substantially between populations: there was little gene flow between Spain and neighbouring populations (*M*<8 migrants per generation), strong bi-directional gene flow between almost all central European populations (*M*>100 for several populations), a tendency for an asymmetric gene flow from eastern Europe to Russia (*M* = 8.3 and 5.3, respectively) and pronounced asymmetric gene flow from Russia to Kazakhstan (*M* = 32.6 and 2.2, respectively; Figure 5a; Table S2). Clade B had present-day and ancestral population sizes of approximately 10,000 and the time since divergence between pairs of populations was approximately 1.5 kyr (Table S2). The gene flow between populations was overall much lower for clade B (several *M*≈1; max *M* = 13.7) than for clade A (several *M*>100), and, if anything, there was weak asymmetric gene flow from southern to northern populations (Figure 5b; Table S2). In a model where all clade A haplotypes were pooled in one population and all B haplotypes in another population, the isolation with migration models produced an estimate of the time since divergence of 18 kyr BP, an ancestral effective population size of 13,000, and present-day effective population sizes of 95,000 and 25,000 for the clade A and B, respectively (Table S2).

## Discussion

### Evidence for two independent postglacial expansions

Our analyses point to the fact that the great reed warbler has expanded along two independent routes from two separated glacial refugia. There are two major clades in the mtDNA sequence tree and a clear geographical pattern in the frequency of these clades still exists among present-day populations. The analyses of mismatch distributions support that two independent expansions have occurred and reject the possibility of one or two large panmictic populations.

The most parsimonious interpretation is that clade B was isolated in a refugium located somewhere in the Middle East and this view gains some support from the analyses of gene flow suggesting low levels of asymmetric gene flow towards north. The location of clade A's refugium is more difficult to track down, because the clade is presently widely dispersed and seems to represent a relatively old expansion. However, two sources of information indicate that the refugium was located in the western parts of the breeding range. First, the gene flow analyses show a slight asymmetric gene flow from Eastern Europe to Western Asia and pronounced asymmetric gene flow from Western Asia to Central Asia. Secondly, a closer inspection of the NJ sequence tree, and the minimum-spanning network, reveals that there is a large, relatively uniform cluster of 26 sequences from Western and Central Asia in the central part of the tree, and no indication that these haplotypes have dispersed to Europe. We conclude that clade A's refugium was located somewhere in South-Western Palaearctic, with a colonisation route that went north of the Black sea to Central Asia. There are several potential, well-known refugia in that region including the Iberian peninsula, Italy and the Balkans, as well as North-Western Africa [1], [25].

The absence of gene flow between the Middle East and Central Asia probably reflects geographical expansion barriers in form of large mountain ridges located between the Black Sea and the Caspian Sea as well as to the east of the Caspian Sea. The great reed warbler is a long-distant migrant where all individuals migrate to Africa south of the Sahara [14], [26]. The migratory routes and overwintering strategies of great reed warblers from Central Asia are unknown, but if these populations descend from European ancestors as our data suggest, their migratory habits might be more similar to those of the European populations, than those of populations in the Middle East [14], [18].

The great reed warbler is highly philopatric in some populations, yet has a relatively high dispersal potential [27]–[29], and it is possible that it is capable of rapidly expanding its range during favourable conditions. Rapid expansions have been documented in a few bird species, e.g. the collared dove (*Streptopelia decaocto*) has colonised much of Europe in only 150 years [30]. Our estimates of the divergence time of the two haplotype clades in great reed warblers range from 18 kyr BP with the IMa method to 65–87 kyr BP based the degree of sequence divergence. These values are within the last glaciation period, which reached its maximum approximately 20 kyr BP [31], [32]. Then it took several thousands of years before the new unoccupied habitat became available. Probably the pristine habitat has been available only during the last few thousand years (maybe up to 10 kyr BP), which sets the maximum date for the expansion. Thus, the estimated expansion dates from the mismatch distribution analyses (i.e. 24 and 20 kyr BP for clade A and clade B, respectively) are too high. The divergence dates between present-day populations estimated with the IMa method seem more plausible: 4 kyr BP for clade A and 1.5 kyr BP for clade B. Still, these estimates are sensitive to mutation rates and generation times, and assume a constant molecular clock and migration-drift equilibrium, thus should be interpreted cautiously. Although it may be very difficult to date the postglacial expansion in the great reed warbler, our data strongly suggest that the expansion of clade A preceded that of clade B. The level of gene flow is higher between populations for clade A, and clade A has colonised a much wider area and is now present all the way from Western Europe to Central Asia.

### Consequences of repeated range expansions and contractions

Speciation may be the most spectacular consequence of repeated glacial cycles, but there are also other important consequences of glacial population bottlenecking and postglacial expansions. For instance, geographical barriers may result in limits to present-day range distributions and failures to colonize seemingly suitable habitat [1], [33]. Thus, glacial range oscillation is one of several factors that contribute to the evolution of species ranges [1], [33]–[35]. The presence of dispersal barriers to the north and north-eastern of the Middle East seems to provide a plausible explanation for the delayed expansion of clade B in great reed warblers.

Postglacial expansions may also leave more subtle traces in form of lower genetic variation in populations at the expanding range limits; referred to as the ‘leading edge hypothesis’ [1], [36]. In the present study, the admixture of the two clades makes the interpretation difficult, but it is true that several populations in the expanding range limit have low levels of haplotype and nucleotide diversity (e.g. the Netherlands and Kazakhstan). However, the two supposedly older populations in the Middle East have low diversity and consequently the leading edge hypothesis does not provide an exclusive explanation to mtDNA diversity in great reed warblers.

A study of the breeding ecology of the great reed warbler in Sweden has been ongoing for more that two decades [15], [37], [38]. An unexpected result from our previous studies is that molecular markers hitchhike with important fitness genes [39], [40], which suggest that there are high levels of linkage disequilibrium in the population [41], [42]. It is well-known that admixture may cause linkage disequilibrium [43], [44] and since admixture may follow from rapid populations expansions and contractions this is yet another important consequence of postglacial range oscillations. Whether postglacial admixture has contributed to the high level of linkage disequilibrium in the great reed warbler ([45], [46]; B. Hansson, K. Csilléry, unpublished) remains to be evaluated, but the present result is suggestive.

The Pleistocene speciation hypothesis suggests that speciation occurs in isolated refugia over one or more full glacial cycles [4]–[6]. The postglacial expansion hypothesis proposes an alternative explanation namely that diversification and speciation takes place during expansion rather than during isolation [10]–[13]. During postglacial expansions the advancing populations may encounter a variety of unoccupied habitats with varying selection regimes, which could drive diversification. In support of this hypothesis, recent divergence with extensive morphological variation has, for example, been documented in several bird species; redpolls (*Carduelis flammea*; [10]; but see [47]), yellow wagtails (*Motacilla flava*; [11]), yellow-rumped warblers (*Dendroica coronata*; [12]) , willow warblers (*Phylloscopus trochilus*; [48]) and dark-eyed juncos (*Junco hyemalis*; [13]).

As mentioned above, great reed warblers in the eastern parts of the range (east of the Black Sea; *A. a. zarudnyi*) have paler plumage than western birds (*A. a. arundinaceus*) [14], [17] and our results suggest that the plumage diversification took place after the last glaciation during the easterly expansion of clade A. This gives support to the postglacial expansion hypothesis and the role of expansions in driving diversification in comparatively short time periods. However, the paler *A. a. zarudnyi* is also recognised in eastern parts of the Middle East [14], [17], i.e. in the region where our Iranian population with clade B haplotypes is located. This suggests a more complicated history of the plumage diversification in the great reed warbler. For instance, the plumage variation may have appeared recently and differentiated independently in each expanding clade. Alternatively, the plumage variation may be of old origin and could have survived in both refugia, with the plumage differentiation following during the expansion of each clade. Both these scenarios are in line with the postglacial expansion hypothesis.

Our data show that the refugia populations became separated during the last glacial period and that the postglacial expansions have resulted in substantial admixture in Central and Eastern Europe. However, there is no indication of any (strong) pre- or post-zygotic isolation mechanisms between the great reed warbler clades ([45], [46]; S. Bensch, B. Hansson, D. Hasselquist, unpublished). This is in line with the Pleistocene speciation hypothesis invoking that much longer periods of time in isolation are needed for speciation to occur [4]–[6].

## Materials and Methods

### Study populations and molecular methods

Samples of great reed warblers were obtained from 15 breeding populations throughout the species' breeding range: from Spain in west to Kazakhstan in east; and from Iran in south to Sweden in north (Table 1). This means that we have covered a substantial part of the breeding range, except from the most north-eastern and eastern parts of the range (Southern Siberia and North-Western Mongolia; [14], [17]).

For all individuals, except for those in the Netherlands, DNA was extracted from blood (5–50 µL stored in SET-buffer) by a standard phenol-chloroform extraction protocol. The samples from the Netherlands consisted of dried contour feathers plucked from nestlings (different nests) and DNA was extracted by using 5% Chelex 100 (Biorad; for references, see [18]).

The mitochondrial control region II in great reed warblers (GenBank: AF111791) was amplified and sequenced with the primers BCML4 (5′-TTCACAGATACAAATGCTTGGG- 3′) and FTPH3 (5′-AAGGCTGGGAGAGTTGTTGA- 3′) following the procedures in Bensch and Hasselquist [18]. These two primers give a product of 577 base-pairs (bps) and previous analyses have confirmed that 494 bps of this fragment are from the control region II and the 81 bps flanking the control region (3′) from the tRNA^Phe^ gene [18]. Maternal inheritance of haplotypes in families and absence of double base calling [18] confirm the mitochondrial (and not NUMT) origin of this fragment.

Polymerase chain reactions (PCRs) were performed in volumes of 25 µL and included 10–50 ng of total genomic DNA, 0.125 mM of each nucleotide, 1.5 mM MgCl_2_, 0.6 µM of each primer and 0.5 U AmpliTaq polymerase. The PCRs were run using the following conditions: 30 s at 94°C, 30 s at 50°C, 30 s at 72°C (35 cycles). Before the cyclic reactions the samples were incubated at 94°C for 2 min, and after completion at 72°C for 10 min. The PCR product was precipitated (NH_4_Ac and ethanol) and then dissolved in 20 µL of water; 2–4 µL was then used for sequencing (BigDye sequencing kit; Applied Biosystems) in an ABI Prism 3100 capillary sequencer (Applied Biosystems).

The data set of 93 control region sequences from six populations gathered and analysed in Bensch and Hasselquist [18] was increased to a total number of 281 sequences from 15 populations (Table 1).

### Statistical methods

Basic population statistics were calculated using the program DnaSP 4.10.9 [49]: haplotype diversity (*H*) and nucleotide diversity (*π*) were calculated according to Nei [50].

Evolutionary relationships between haplotypes were assessed by the Neighbour-Joining method with MEGA 3.1 [51]. We used Tamura and Nei's [52] distance measure and a gamma correction parameter alpha of 0.04 to account for among-site variation in evolutionary rate [53], as had been estimated previously with Puzzle [54] (see [18]). The phylogeny was tested with the Bootstrap procedure (10,000 replications). We also conducted a phylogenetic analysis using the program MrBayes 3 [55], implementing a GTR+I+G model of molecular evolution as first selected by the program MrModeltest2 [56]. The Bayesian phylogeny was obtained by using four heated and one cold MCMC chain, which was sampled every 200 generations over 20 million generations generating 100,000 trees. The first 25% of the trees were discarded as the burn-in period, and the remaining 75,000 trees were used to construct a majority consensus tree. The result from the Bayesian analysis was qualitatively similar to that of the Neighbour-Joining method, with a strong support (100%) for the branching of the two major clades (data not shown; cf. Figure S1).

We calculated the degree of genetic differentiation measured as *F*_ST_ (following [57], equation 3) between pairs of populations in DnaSP. Neighbour-Joining population trees were calculated in MEGA using these pair-wise *F*_ST_-values. Minimum spanning networks were drawn in Network 4.2.0.1 (http://www.fluxus-technology.com/).

The average number of nucleotide substitutions per site between the clades, *D*_XY_, and the number of net nucleotide substitutions per site between clades, *D*_A_, were calculated in DnaSP (following [50], equation 10.20 and 10.21, respectively). Standard errors of *D*_XY_ and *D*_A_ were calculated in MEGA using the Bootstrap procedure with 1000 replicates. These parameters were used to estimate the time since divergence of the two clades with the expressions *t* = (*D*_XY_/2*μ*)×*g*, and *t* = (*D*_A_/2*μ*)×*g*, respectively, where *μ* is the rate of substitution per site Myr^−1^ and *g* is the generation time (2 years; [18]). The rate of molecular evolution of the avian control region is uncertain and we use a value of 15% but also provide the results for a range of 10–20% per site per million years (s/s/Myr) [13], [21]–[24].

We calculated mismatch distributions in DnaSP. The observed mismatch distribution was tested against expected values in a stable population (i.e. population with constant population size; following [58]), and in a growing or declining population (following [59], equation 4), with χ^2^-tests (pooling classes to achieve a minimum of 5 expected counts in each class). We estimated the parameter *τ*, i.e. the date of the expansion measured in units of mutational time, which can be used to estimate the time since expansion with the expression *t* = (*τ*/2*μk*)×*g*, where *μ* and *g* are as above and *k* is the sequence length (i.e. 494 bp).

We also inferred populations sizes, asymmetric gene flow and time since divergence between pairs of populations with demographic population genetic models implemented in the program IMa, Isolation with Migration, analytic, version 7/13/2007 [19], [20]. We did preliminary runs with different settings to determine a suitable multi-dimensional parameter space; we started with default values and increased them gradually to achieve output parameters within the parameter space. In the final runs, we used a value of 100 for the scalars of *θ* of all populations (i.e. population 1, population 2 and the ancestral population); a value of 100 as the maximum migration rate between populations (from population 1 to population 2, and from population 2 to population 1, respectively); and 10 for the maximum time of population splitting. For each pair of populations we used a ‘burn-in’ period of 1×10^6^ steps and a sampling period of 1×10^7^ steps. The output parameters (*θ*_1_, *θ*_2_, *θ*_A_, *m*_1_, *m*_2_, *t*) were expressed in demographic units as follow: effective population size, *N*_E_ = *θ*/4*μkg*; number of migrants per generation, *M* = 2*N*_E_×(*m*×2*μk*) = *θ*×*m*/2*g*; divergence time in years, *T* = *tg*/2*μk*; where *μ*, *k* and *g* are as above.

## Supporting Information

Table S1 F_ST_-values between populations using data from (a) clade A and B haplotypes; (b) clade A haplotypes; and (c) clade B haplotypes.
(0.08 MB DOC)

Table S2 Estimates of demographic parameters from two-population isolation with migration models implemented in the program IMa [19], [20].
(0.08 MB DOC)

Figure S1 Neighbour-Joining tree based on 281 mtDNA control region II sequences of great reed warblers from 15 breeding populations. Labels refer to population (SPA-Spain; NET-the Netherlands; GER-Germany; SWE-Sweden; LAT-Latvia; BEL-Belarus; UKR-Ukraine; CZE-the Czech Republic; HUN-Hungary; BUL-Bulgaria; GRE-Greece; RUS-Russia; KAZ-Kazakhstan; TUR-Turkey; IRA-Iran). The two major haplotype clades are shown, clade A and B. The dotted line indicates a relatively large uniform cluster of sequences within clade A only found in Russia and Kazakhstan (see text for details).
(3.30 MB TIF)
