# Supplementary material for: Postglacial Colonisation Patterns and the Role of Isolation and Expansion in Driving Diversification in a Passerine Bird
Source: PLoS One. 2008 Jul 30;3(7):e2794. doi: 10.1371/journal.pone.0002794 (PMC2467487; doi:10.1371/journal.pone.0002794)
Supplement: Table S1 — FST-values between populations using data from (a) clade A and B haplotypes; (b) clade A haplotypes; and (c) clade B haplotypes. (0.08 MB DOC) [file pone.0002794.s001.doc]

| ***(a) Clade A and B*** | Spa | Net | Swe | | Lat | | Ger | | Cze | | Hun | Bel | | Ukr | | Rus | | Kaz | Gre | | | Bul | Tur | Ira |
| --- | --- | --- | --- | --- | --- | --- | --- | --- | --- | --- | --- | --- | --- | --- | --- | --- | --- | --- | --- | --- | --- | --- | --- | --- |
| Spain | (11) |  |  | |  | |  | |  | |  |  | |  | |  | |  |  | | |  |  |  |
| Netherlands | -0.038 | (10) |  | |  | |  | |  | |  |  | |  | |  | |  |  | | |  |  |  |
| Sweden | 0.030 | 0.049 | (22) | |  | |  | |  | |  |  | |  | |  | |  |  | | |  |  |  |
| Latvia | 0.127 | 0.146 | 0.004 | | (20) | |  | |  | |  |  | |  | |  | |  |  | | |  |  |  |
| Germany | 0.078 | 0.071 | -0.004 | | -0.018 | | (19) | |  | |  |  | |  | |  | |  |  | | |  |  |  |
| Czech | 0.213 | 0.230 | 0.068 | | -0.009 | | 0.033 | | (17) | |  |  | |  | |  | |  |  | | |  |  |  |
| Hungary | 0.150 | 0.123 | 0.035 | | 0.027 | | -0.017 | | 0.080 | | (20) |  | |  | |  | |  |  | | |  |  |  |
| Belarus | 0.180 | 0.193 | 0.031 | | -0.029 | | 0.005 | | -0.031 | | 0.050 | (17) | |  | |  | |  |  | | |  |  |  |
| Ukraine | 0.233 | 0.254 | 0.083 | | -0.014 | | 0.036 | | -0.047 | | 0.090 | -0.028 | | (18) | |  | |  |  | | |  |  |  |
| Russia | 0.047 | 0.058 | 0.005 | | 0.010 | | -0.004 | | 0.074 | | 0.066 | 0.038 | | 0.076 | | (28) | |  |  | | |  |  |  |
| Kazakhstan | 0.349 | 0.284 | 0.297 | | 0.290 | | 0.211 | | 0.326 | | 0.275 | 0.323 | | 0.339 | | 0.191 | | (35) |  | | |  |  |  |
| Greece | 0.198 | 0.211 | 0.070 | | 0.005 | | 0.030 | | -0.032 | | 0.072 | -0.019 | | -0.020 | | 0.063 | | 0.281 | (20) | | |  |  |  |
| Bulagria | 0.319 | 0.334 | 0.148 | | 0.039 | | 0.095 | | -0.030 | | 0.135 | -0.002 | | -0.030 | | 0.152 | | 0.413 | 0.003 | | | (20) |  |  |
| Turkey | 0.772 | 0.778 | 0.630 | | 0.510 | | 0.564 | | 0.341 | | 0.609 | 0.434 | | 0.377 | | 0.617 | | 0.773 | 0.347 | | | 0.317 | (17) |  |
| Iran | 0.867 | 0.871 | 0.721 | | 0.599 | | 0.655 | | 0.417 | | 0.702 | 0.521 | | 0.456 | | 0.710 | | 0.864 | 0.446 | | | 0.382 | 0.126 | (7) |
| ***(b) Clade A*** | | Spa | | Net/Ger | | | | Cze/Hun | | Swe/Lat | | | Bel/Ukr | | | | Gre/Bel/Tur | | | Rus | | | Kaz | |
| Spain | | (11) | |  | | | |  | |  | | |  | | | |  | | |  | | |  | |
| Netherlands/Germany | | 0.027 | | (10/16) | | | |  | |  | | |  | | | |  | | |  | | |  | |
| Czech/Hungary | | 0.127 | | 0.022 | | | | (10/18) | |  | | |  | | | |  | | |  | | |  | |
| Sweden/Latvia | | 0.003 | | -0.002 | | | | 0.046 | | (19/15) | | |  | | | |  | | |  | | |  | |
| Belarus/Ukraine | | 0.029 | | -0.021 | | | | 0.013 | | -0.008 | | | (11/11) | | | |  | | |  | | |  | |
| Greece/Bulgaria/Turkey | | 0.047 | | -0.006 | | | | 0.004 | | 0.019 | | | -0.012 | | | | (13/10/1) | | |  | | |  | |
| Russia | | 0.027 | | 0.022 | | | | 0.148 | | 0.047 | | | 0.034 | | | | 0.074 | | | (23) | | |  | |
| Kazakhstan | | 0.349 | | 0.217 | | | | 0.322 | | 0.311 | | | 0.254 | | | | 0.255 | | | 0.165 | | | (35) | |
| ***(c) Clade B*** | | Ger/Cze/Hun | | | | Swe/Lat | | | | Bel/Ukr/Rus | | | | | Gre/Bul | | | | | | Tur/Ira | | | |
| Germany/Czech/Hungary | | (3/7/2) | | | |  | | | |  | | | | |  | | | | | |  | | | |
| Sweden/Latvia | | 0.022 | | | | (3/5) | | | |  | | | | |  | | | | | |  | | | |
| Belarus/Ukraine/Russia | | 0.022 | | | | 0.054 | | | | (6/7/5) | | | | |  | | | | | |  | | | |
| Greece/Bulgaria | | -0.059 | | | | 0.065 | | | | 0.052 | | | | | (7/10) | | | | | |  | | | |
| Turkey/Iran | | 0.036 | | | | 0.194 | | | | 0.238 | | | | | 0.033 | | | | | | (16/7) | | | |

Closely located populations were pooled in (b) and (c). Sample sizes are given in the diagonal.
