# Supplementary material for: Postglacial Colonisation Patterns and the Role of Isolation and Expansion in Driving Diversification in a Passerine Bird
Source: PLoS One. 2008 Jul 30;3(7):e2794. doi: 10.1371/journal.pone.0002794 (PMC2467487; doi:10.1371/journal.pone.0002794)
Supplement: Table S2 — Estimates of demographic parameters from two-population isolation with migration models implemented in the program IMa [19], [20]. (0.08 MB DOC) [file pone.0002794.s002.doc]

| Population 1 | Population 2 | Output parameter | | | | | | Parameters in demographic units | | | | | |
| --- | --- | --- | --- | --- | --- | --- | --- | --- | --- | --- | --- | --- | --- |
|  |  | *θ*_1_ | *θ*_2_ | *θ*_A_ | *m*_1_ | *m*_2_ | *t* | *N*E_1^#^ | *N*E_2^#^ | NE_A^#^ | *M*_1_^¤^ | *M*_2_^¤^ | *T*^$^ |
| ***Clade A*** |  |  |  |  |  |  |  |  |  |  |  |  |  |
| Spain | Netherlands/Germany | 11.99 | 12.78 | 3.82 | 1.65 | 1.15 | 0.37 | 20225 | 21558 | 6445 | 4.95 | 3.67 | 4926 |
| Spain | Czech/Hungary | 9.76 | 114.82 | 5.50 | 3.15 | 0.25 | 0.32 | 16459 | 193696 | 9277 | 7.68 | 7.18 | 4251 |
| Netherlands/Germany | Sweden/Latvia | 37.00 | 40.28 | 3.83 | 4.95 | 2.75 | 0.39 | 62420 | 67955 | 6457 | 45.79 | 27.69 | 5196 |
| Netherlands/Germany | Czech/Hungary | 47.99 | 394.81 | 8.89 | 45.35 | 37.85 | 0.25 | 80960 | 666003 | 14993 | 544.12 | 3735.86 | 3306 |
| Sweden/Latvia | Belarus/Ukraine | 41.97 | 33.26 | 3.66 | 0.45 | 4.95 | 0.31 | 70802 | 56113 | 6170 | 4.72 | 41.16 | 4116 |
| Czech/Hungary | Belarus/Ukraine | 37.57 | 33.15 | 7.03 | 20.15 | 2.35 | 0.29 | 63383 | 55926 | 11863 | 189.28 | 19.48 | 3846 |
| Czech/Hungary | Greece/Bulgaria/Turkey | 51.51 | 46.04 | 7.36 | 24.65 | 99.95 | 0.37 | 86887 | 77667 | 12412 | 317.41 | 1150.45 | 4926 |
| Belarus/Ukraine | Greece/Bulgaria/Turkey | 12.92 | 12.20 | 8.19 | 10.20 | 31.15 | 0.25 | 21801 | 20573 | 13818 | 32.96 | 94.97 | 3306 |
| Belarus/Ukraine | Russia | 6.45 | 10.29 | 6.72 | 5.15 | 2.05 | 0.17 | 10879 | 17360 | 11342 | 8.30 | 5.27 | 2227 |
| Russia | Kazakhstan | 5.54 | 4.44 | 5.12 | 23.55 | 1.95 | 0.46 | 9353 | 7496 | 8635 | 32.64 | 2.17 | 6140 |
|  | *Mean* | *26.27* | *70.21* | *6.01* | *13.93* | *18.44* | *0.31* | *44317* | *118435* | *10141* | *118.79* | *508.79* | *4224* |
|  | *Median* | *24.96* | *33.21* | *6.11* | *7.68* | *2.55* | *0.31* | *42111* | *56020* | *10309* | *32.80* | *23.59* | *4184* |
|  |  |  |  |  |  |  |  |  |  |  |  |  |  |
| ***Clade B*** |  |  |  |  |  |  |  |  |  |  |  |  |  |
| Germany/Czech/Hungary | Sweden/Latvia | 4.82 | 2.96 | 5.16 | 0.05 | 1.45 | 0.04 | 8131 | 4993 | 8701 | 0.06 | 1.07 | 472 |
| Germany/Czech/Hungary | Belarus/Ukraine/Russia | 5.57 | 5.79 | 5.79 | 0.35 | 0.05 | 0.13 | 9389 | 9772 | 9772 | 0.49 | 0.07 | 1687 |
| Germany/Czech/Hungary | Greece/Bulgaria | 3.59 | 4.59 | 7.41 | 0.45 | 6.45 | 0.01 | 6057 | 7744 | 12500 | 0.40 | 7.40 | 67 |
| Sweden/Latvia | Belarus/Ukraine/Russia | 0.28 | 4.13 | 3.21 | 0.45 | 13.25 | 0.19 | 464 | 6963 | 5415 | 0.03 | 13.67 | 2497 |
| Belarus/Ukraine/Russia | Greece/Bulgaria | 8.64 | 5.37 | 5.34 | 4.45 | 4.75 | 0.12 | 14582 | 9060 | 9012 | 9.62 | 6.38 | 1552 |
| Greece/Bulgaria | Turkey/Iran | 4.54 | 5.21 | 4.03 | 2.25 | 4.95 | 0.27 | 7656 | 8787 | 6801 | 2.55 | 6.45 | 3576 |
|  | *Mean* | *4.57* | *4.68* | *5.16* | *1.33* | *5.15* | *0.12* | *7713* | *7886* | *8700* | *2.19* | *5.84* | *1642* |
|  | *Median* | *4.68* | *4.90* | *5.25* | *0.45* | *4.85* | *0.12* | *7893* | *8265* | *8857* | *0.45* | *6.41* | *1619* |
|  |  |  |  |  |  |  |  |  |  |  |  |  |  |
| ***Clade A and B*** |  |  |  |  |  |  |  |  |  |  |  |  |  |
| Clade A, all populations | Clade B, all populations | 56.27 | 14.59 | 7.44 | 0.05 | 0.05 | 1.37 | 95051 | 24643 | 12573 | 0.70 | 0.18 | 18446 |

Models for clade A and clade B haplotypes were run separately to evaluate gene flow between pairs of populations for each clade. Data from all populations were used to estimate effective population sizes and divergence time between clade A and B.

# Effective population size: *θ*/4*µkg*, where *µ* is the rate of substitution per site Myr^-1^, *k* the sequence length and *g* is the generation time.

¤ Number of migrants per generation: *M*_1_ = 2*N*E_1×(*m*_1_×2*µk*) = *θ*_1_×*m*_1_/2*g*; *M*_2_ = 2*N*E_2×(*m*_2_×2*µk*) = *θ*_2_×*m*_2_/2*g*

$ Divergence time in years: *T* = *tg*/2*µk*
