# Supplementary figures and images for: Postglacial Colonisation Patterns and the Role of Isolation and Expansion in Driving Diversification in a Passerine Bird
Source: PLoS One. 2008 Jul 30;3(7):e2794. doi: 10.1371/journal.pone.0002794 (PMC2467487; doi:10.1371/journal.pone.0002794)

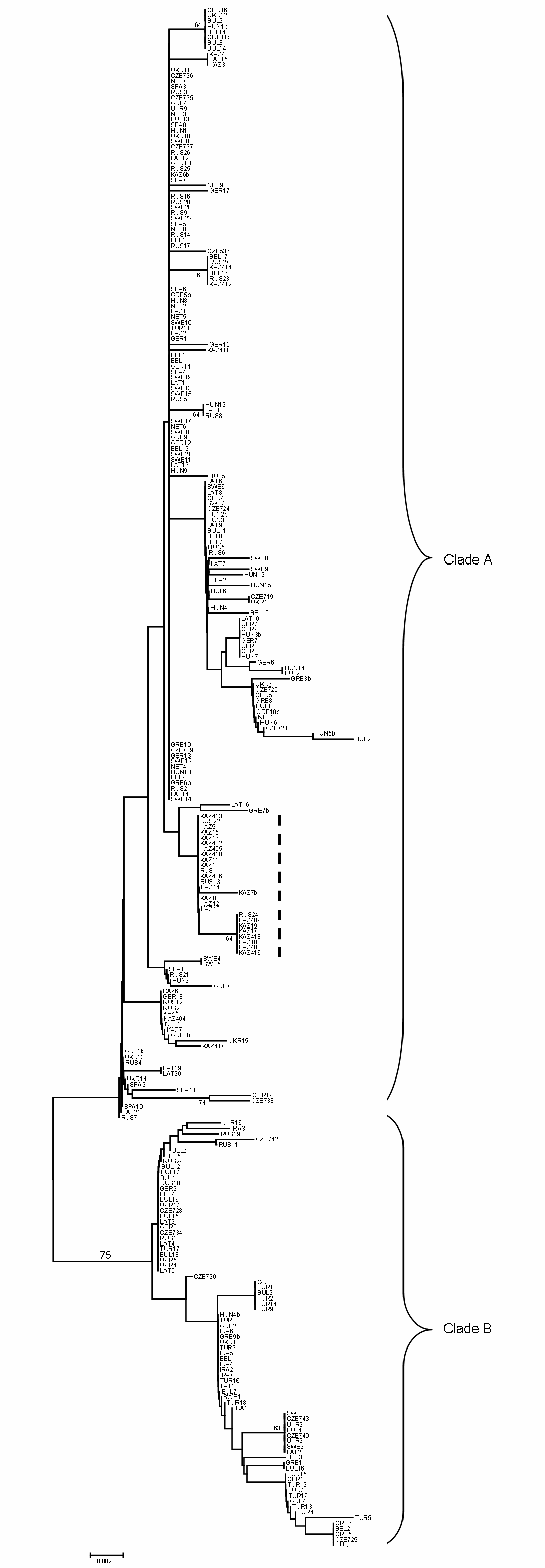

Supplement: Figure S1 — Neighbour-Joining tree based on 281 mtDNA control region II sequences of great reed warblers from 15 breeding populations. Labels refer to population (SPA-Spain; NET-the Netherlands; GER-Germany; SWE-Sweden; LAT-Latvia; BEL-Belarus; UKR-Ukraine; CZE-the Czech Republic; HUN-Hungary; BUL-Bulgaria; GRE-Greece; RUS-Russia; KAZ-Kazakhstan; TUR-Turkey; IRA-Iran). The two major haplotype clades are shown, clade A and B. The dotted line indicates a relatively large uniform cluster of sequences within clade A only found in Russia and Kazakhstan (see text for details). (3.30 MB TIF) [file pone.0002794.s003.tif]
